# How do trial teams plan for retention during the design stage of the trial? A scoping review protocol

**DOI:** 10.1186/s13063-022-06866-w

**Published:** 2022-11-17

**Authors:** Ellen Murphy, Katie Gillies, Frances Shiely

**Affiliations:** 1 Trials Research and Methodologies Unit (TRAMS), Health Research Board Clinical Research Facility University College Cork, Cork, Ireland; 2 Health Services Research Unit, University of Aberdeen, Aberdeen, UK; 3 School of Public Health, University College Cork, Cork, Ireland

**Keywords:** Scoping review, Randomised controlled trial protocols, Retention strategies, Trial design stage

## Abstract

**Background:**

Retention remains a major challenge for many clinical trials. The SPIRIT guidelines state the following information on retention should be included in the trial protocol “Plans to promote participant retention and complete follow-up, including list of any outcome data to be collected for participants who discontinue or deviate from intervention protocols”. This guidance shows the importance of planning retention methods and handling missing data as this can impact how the results of the trial are interpreted. The most recent Cochrane review of strategies to improve retention in clinical trials highlighted that some trials implemented multiple retention strategies and we questioned whether the use of multiple strategies was planned at the design stage and included in the protocol or are strategies implemented when retention becomes an issue within the trial. The purpose of our scoping review is to establish if and how trial teams prepare for retention at the design phase of clinical trials.

**Methods and analysis:**

We will follow the methodological framework and guidelines for scoping reviews outlined by the Joanna Briggs Institute. We will search MEDLINE/PubMed, Scopus, EMBASE, CINAHL (EBSCO), and Web of Science. A comprehensive search strategy for PubMed was developed in collaboration with an experienced research librarian. We will include protocols for phase 2, 3, and 4 RCTs as well as pilot and feasibility studies. The screening process will involve two reviewers. EM will independently screen all titles and abstracts. FS will screen 10% of the overall search output, and where necessary full protocol texts will be screened to determine eligibility. We will randomly sample eligible protocols to ensure the protocols represent a variety of trial and intervention types. Data will be extracted from each protocol and the results will be synthesised. The analysis will be qualitative using a narrative summary and descriptive statistics where appropriate.

**Discussion:**

The scoping review will help trial methodologists better understand if retention strategies are planned for during the design stage of the trial contributing to the PRioRiTy II unanswered question “How should people who run trials plan for retention during their funding application and creation of the trial (protocol development)?”.

**Supplementary Information:**

The online version contains supplementary material available at 10.1186/s13063-022-06866-w.

## Background/rationale

Randomised controlled trials (RCTs) are conducted in accordance with a trial protocol. Having a protocol that is incomplete and non-transparent makes it difficult to critically appraise the trial [1]. Protocols are needed for the readers of the corresponding paper to be able to fully appraise and interpret the results of the trial [2]. As per the 2013 SPIRIT (Standard Protocol Items: Recommendations for Interventional Trials) definition, a protocol is “a document that provides sufficient detail to enable understanding of the background, rationale, objectives, study population, interventions, methods, statistical analyses, ethical considerations, dissemination plans, and administration of the trial, replication of key aspects of trial methods and conduct; and appraisal of the trial’s scientific and ethical rigour from ethics approval to dissemination of results” (3;202). Prior to the introduction of SPIRIT, the content and quality of protocols differed greatly [3, 4], but the 33-item SPIRIT checklist for the minimum recommended protocol items improved this. Having a clearly written protocol increases transparency in trial conduct. Protocols are usually published prior to the trial paper publication [5]. However, this is not always the case, and some protocol publications will require payment for access. For example, of the cancer clinical trials published in January of 2020 (*n* = 113), only 11.3% had a publicly accessible protocol that was not behind a paywall [2]. This further limits transparency and hinders replication in trial methods and conduct, which has been recommended for trial retention strategies in order to improve the evidence base for their effectiveness [6].

Retention remains a major challenge for many clinical trials [7]. The SPIRIT guidelines recommend the following information on retention be included in the trial protocol in Sect. 18b “Plans to promote participant retention and complete follow-up, including list of any outcome data to be collected for participants who discontinue or deviate from intervention protocols” (8;3). These of course are minimum requirements but the information can impact how missing data is dealt with and interpreted in the analysis of the trial [8].

A recent Cochrane systematic review of strategies to improve retention in clinical trials found that there were no strategies for which the quality of evidence was high that showed improved retention. The review also highlighted that some trials implement multiple retention strategies [6], and thus we questioned whether the use of multiple strategies is planned at the design stage and included in the protocol or are strategies implemented when retention becomes an issue within the trial. Evidence from a small number of interviews with trial staff is variable [9]. Prospective retention planning informs appropriate costing and implementation of retention strategies but also increases transparency in trial conduct.

## Objective

The purpose of our scoping review is to establish if and how trial teams prepare for retention at the design phase of clinical trials. This will contribute to the evidence base for the PRioRiTy II (Prioritising Retention in Randomised Trials) unanswered question “How should people who run trials plan for retention during their funding application and creation of the trial (protocol development)?” [10].

## Methods

The scoping review will be conducted in accordance with the guidelines and framework outlined by the Joanna Briggs Institute (JBI) [11], the most recent framework for scoping reviews which builds on prior work [12–14]. The framework consists of the following nine steps:

1. Defining and aligning the objective/s and question/s.
2. Developing and aligning the inclusion criteria with the objective/s and question/s.
3. Describing the planned approach to evidence searching, selection, data extraction and presentation of the evidence.
4. Searching for the evidence.
5. Selecting the evidence.
6. Extracting the evidence.
7. Analysis of the evidence.
8. Presentation of the results.
9. Summarising the evidence in relation to the purpose of the review, making conclusions and noting any implications of the findings.

The guidance from the Joanna Briggs Institute [11] along with the newly developed reporting guidelines for scoping reviews: the Preferred Reporting Items for a Systematic Review and Meta-Analysis Protocols Extension for Scoping Reviews (PRISMA-ScR) [15] has been consulted in the development of this protocol (Additional File 1).

### Data sources and search strategy

A comprehensive search strategy for PubMed was developed in collaboration with an experienced research librarian at University College Cork and is shown below. The following electronic databases will be searched for relevant protocols, MEDLINE/PubMed, Scopus, EMBASE, CINAHL (EBSCO), and Web of Science. The search will be adapted as appropriate for each database using the software Polyglot which translates search strategies across databases. The search and screening process will take place over a six-week period.

### Example of PubMed search

(“randomised controlled trial”[Title/Abstract]) OR (“randomized controlled trial”[Title/Abstract])) OR (“randomised clinical trial”[Title/Abstract])) OR (“randomized clinical trial”[Title/Abstract])) OR (“randomized controlled trials as topic”[MeSH Terms])) AND (“protocol”[Title/Abstract])) OR (“clinical trial protocols as topic”[MeSH Terms]).

This is the full search strategy developed for PubMed. The limits that will be applied include published in the English language between the years of 2014 and 2019 (inclusive). No other limits will be applied to the search.

### Inclusion criteria

#### Population

The population include the following: protocols of RCTs that include adults and/or children of any age.

#### Concepts

The concepts includes the following: protocols of RCTs investigating any treatment/intervention type for any disease area. Randomisation can be at the cluster or individual level. The concepts also include protocols of RCTs investigating any comparator including placebo and examining any outcome.

#### Context

The context of this scoping review is open sources of evidence pertaining to any contextual setting.

#### Types of evidence sources

We will include trial protocols published in the English language. We will include protocols from 2014 to 2019 (inclusive), giving sufficient time to see the effect of the SPIRIT guidelines, which were published in 2013. We will include protocols for phase 2, 3, and 4 RCTs as well as pilot and feasibility studies.

#### Screening and selection process

EM will systematically collate and import titles and abstracts of all electronically sourced search results to EndNote, grouping results separately for each database. Duplicates will be removed, and the remaining results will be exported to the Rayyan QCRI software for screening. The screening process will involve two reviewers (EM and FS). EM will independently screen all titles and abstracts. FS will screen a random selection of 10% of the overall search output. If there is less than 80% agreement on the random 10% of the search output that is double screened, we will undertake screening of a further 10% validation sample. We will reiterate this until > 80% agreement on the validation sample is reached. If disagreement arises between these two reviewers regarding the eligibility of a protocol, a third reviewer KG will be consulted, and where necessary full protocol texts will be screened to determine eligibility. The protocol screening and selection process will be presented both narratively and graphically using a PRISMA (Transparent Reporting of Systematic Reviews and Meta-Analyses) flow diagram [16]. Details of excluded sources at full-text review will be outlined and included in the review with reasons for the protocol exclusion.

#### Sampling

We will select a random sample of 10% of eligible protocols for data extraction; the random sample will be chosen by including blocks of 10 protocols from the list of eligible protocols for inclusion. A randomly chosen 10% will provide a sufficient representation of all eligible protocols.

#### Data management and data charting (extraction) process

Following screening for eligibility all data on retention plans will be extracted. Data to be extracted is outlined in Table 1. The main outcome of interest is whether SPIRIT item 18b is satisfied (Table 1, variable 7), since this statement has sub-requirements during the data charting process we will divide the statement into three aspects “plans to promote participant retention” and “plans to complete follow-up, including list of any outcome data to be collected for participants who discontinue from intervention protocols” and “plans to complete follow-up, including list of any outcome data to be collected for participants who deviate from intervention protocols”. Dividing the statement into sub-statements will more accurately reflect whether SPIRIT item 18b is satisfied or not. With regard to retention strategies we specifically mean an action/activity that is conducted with the purpose of reducing missing data/improving data completeness, we will not include activities to improve adherence or compliance to an intervention. Where there is ambiguity on the purpose of specific actions, i.e. if they are for participant retention or not, the protocol authors will be contacted. Where contact is not possible/a reply is not received, the ambiguous information will be highlighted and included in the data extraction. Prior to the full data charting process, the data extraction form will be piloted using a sample of 10 protocols as the variables to be extracted may need to be refined and improved to best meet the objectives of the scoping review. Data charting will be carried out by one reviewer (EM) and a random sample (10%) of the protocols will be double extracted and checked for consistency by (FS) to ensure consistency and improve the reliability of the data extraction process. If there is less than 80% agreement regarding the data extracted in the 10% random sample of protocols, we will undertake data extraction of a further 10% validation sample. We will reiterate this until > 80% agreement on the validation sample is reached. Data charting will be conducted, and information will be entered into a Microsoft Excel file.

### Data items

**Table 1:** Variables to be extracted

Variables to be extracted
1. .Protocol title, author and year, source of funding
2. Trial characteristics (disease area, patient population, duration of the follow-up period, number of follow-up assessments, intervention, and comparator)
3. Primary outcome (the primary outcome, whether it is a patient-reported vs non-patient-reported outcome)
4. Planned sample size
5. Mention of using the SPIRIT guidelines in the development of the protocol (“yes” or “no”)
6. Description of retention strategies outlined in the protocol
7. Does the description of the retention strategy include all the information recommended by the SPIRIT guidelines Sect. 18b (“yes” or “no”) “Plans to promote participant retention and complete follow-up including list of any outcome data to be collected for participants who discontinue or deviate from intervention protocols” (8;3)
8. Mode of follow-up, e.g. questionnaire, clinic visit, telephone call, etc
9. Routine data collection (yes or no)
10. Mention of Patient and Public Involvement (PPI) in relation to retention
11. Mention of cost associated with retention strategy

#### Data synthesis

We will synthesise the data in a narrative synthesis with descriptive statistics where appropriate. The descriptive statistics that will be generated will include the frequencies and percentages of the trial characteristics such as the percentage of trials conducted in different disease areas, percentage of trials conducted among vulnerable populations, percentage of trials that are publicly or privately funded, and the percentage of drug trials, surgical trials, non-drug/behavioural intervention, and medical device trials, modes of follow-up methods used by trials, and number of trials with patient-reported primary outcomes vs non-patient-reported outcomes. We will also generate the percentage of trial protocols that have satisfied the SPIRIT item 18b statement.

Since we will include pilot and feasibility trial protocols, we plan to conduct a sub-analysis of this group of studies as one of the purposes of pilot and feasibility trials may be to develop and test retention strategies.

The content will be analysed to determine if the protocol complies with the SPIRIT guidelines for retention strategies Sect. 18b- “Plans to promote participant retention and complete follow-up including list of any outcome data to be collected for participants who discontinue or deviate from intervention protocols” (8;3). We will divide the statement into three aspects “plans to promote participant retention” and “plans to complete follow-up including list of any outcome data to be collected for participants who discontinue from intervention protocols” and “plans to complete follow-up including list of any outcome data to be collected for participants who deviate from intervention protocols”. This will be recorded as either “yes” or “no” for each separate aspect. If the protocols include information regarding planned retention strategies, this information will be analysed in the narrative synthesis. The narrative synthesis approach will be based on the Guidance on the Conduct of Narrative Synthesis in Systematic Reviews [17].

### Presentation of findings

We will present the search results in a PRISMA flow diagram illustrating the total number of protocols generated by the search strategy and the number of protocols excluded following the application of the inclusion/exclusion criteria and ultimately the number of protocols included in the scoping review. Summary tables will depict the study characteristics described in the included protocols. Additional tables, figures, and narrative descriptions will illustrate the data addressing our research question. The findings of the scoping review will be disseminated via publication in a peer-reviewed journal.

## Discussion

Conducting a scoping review of randomised controlled trial protocols to investigate if retention strategies are planned by trial teams during the design stage of the trial will contribute to the PRioRiTy II unanswered question “How should people who run trial plan for retention during their funding application and creation of the trial (protocol development)?” [10]. The strength of this review is the intention to include a variety of trial types as described in the methods section. The review will highlight the gaps that exist in the planning and communicating of retention strategies in clinical trial protocols, which may have the potential to increase transparency in trial conduct and make trials more efficient as it will highlight to researchers the lack of consideration and/or communication of trial retention strategies during the initial planning and design stages of the trial. Foreplaning could increase retention strategy success as trial teams will be able to spend greater time researching the most effective strategies in terms of retaining participants and carefully consider the resources required as retention strategies can be very expensive to implement often with little reward in terms of retaining participants [6, 18, 19]. The findings of this review have the potential to inform trialists to plan retention strategies during the design stage of the trial rather than when retention issues appear. The review will also help identify questions for future research projects and findings from this review can be explored in more depth in future research investigating retention strategies in clinical trials.

## Supplementary Information

**Additional file 1.** 

## Data Availability

Not applicable as this is a protocol for a scoping review.

## Abbreviations

RCTs: Randomised controlled trials
SPIRIT: Standard Protocol Items: Recommendations for Interventional Trials
PRioRity: Prioritising Retention in Randomised Controlled Trials
PRISMA-ScR: Preferred Reporting Items for a Systematic Review and Meta-Analysis Protocols Extension for Scoping Reviews
PRISMA: Transparent Reporting of Systematic Reviews and Meta-Analyses
